# Schisandrae Fructus ethanol extract ameliorates inflammatory responses and articular cartilage damage in monosodium iodoacetate-induced osteoarthritis in rats

**DOI:** 10.17179/excli2017-119

**Published:** 2017-03-14

**Authors:** Jin-Woo Jeong, Jongsik Kim, Eun Ok Choi, Da Hye Kwon, Gyu Min Kong, Il-Whan Choi, Bum Hoi Kim, Gi-Young Kim, Ki Won Lee, Ki Young Kim, Sung Goo Kim, Young Whan Choi, Su Hyun Hong, Cheol Park, Yung Hyun Choi

**Affiliations:** 1 Department of Biochemistry, Dongeui University College of Korean Medicine, Busan 614-052, Republic of Korea; 2 Anti-Aging Research Center, Dongeui University, Busan 614-714, Republic of Korea; 3 Department of Anatomy, Kosin University College of Medicine, Busan 602-702, Republic of Korea; 4 Department of Orthopaedic Surgery, College of Medicine, Inje University, Busan, 47392, Republic of Korea; 5 Department of Microbiology, College of Medicine, Inje University, Busan, 47392, Republic of Korea; 6 Department of Anatomy, Dongeui University College of Korean Medicine, Busan 614-052, Republic of Korea; 7 Laboratory of Immunobiology, Department of Marine Life Sciences, Jeju National University, Jeju, 690-756, Republic of Korea; 8 Research Institute, Bio-Port Korea INC, MarineBio-industry Development Center, Busan 619-912, Republic of Korea; 9 Department of Horticultural Bioscience, College of Natural Resource and Life Sciences, Pusan National University, Miryang 627-706, Republic of Korea; 10 Department of Molecular Biology, College of Natural Sciences and Human Ecology, Dongeui University, Busan 614-714, Republic of Korea

**Keywords:** Schisandrae Fructus, osteoarthritis, MIA, inflammatory responses, cartilage degradation

## Abstract

Schisandrae Fructus, the fruit of *Schisandra chinensis* (Turcz.) Baill., is widely used in traditional medicine for the treatment of a number of chronic diseases. Although, Schisandrae Fructus was recently reported to attenuate the interleukin (IL)-1β-induced inflammatory response in chondrocytes in vitro, its protective and therapeutic potential against osteoarthritis (OA) in an animal model remains unclear. Therefore, we investigated the effects of the ethanol extract of Schisandrae Fructus (SF) on inflammatory responses and cartilage degradation in a monosodium iodoacetate (MIA)-induced OA rat model. Our results demonstrated that administration with SF had a tendency to attenuate MIA-induced damage of articular cartilage as determined by a histological grade of OA. SF significantly suppressed the production of pro-inflammatory cytokines such as interleukin (IL)-1β, IL-6, and tumor necrosis factor-α in MIA-induced OA rats. SF also effectively inhibited expression of inducible nitric oxide (NO) synthase and cyclooxygenase-2, thereby inhibiting the release of NO and prostaglandin E_2_. In addition, the elevated levels of matrix metalloproteinases-13 and two biomarkers for diagnosis and progression of OA, such as cartilage oligomeric matrix protein and C-telopeptide of type II collagen, were markedly ameliorated by SF administration. These findings indicate that SF could be a potential candidate for the treatment of OA.

## Introduction

Osteoarthritis (OA) is the most common musculoskeletal disease causing chronic pain and joint disability. OA is characterized by the loss of articular cartilage, involving increased subchondral bone remodeling, osteophyte formation, weakening of the periarticular muscles, and thickening of the capsule and synovial membrane, which lead to functional joint limitations (Guilak, 2011[17]; Mobasheri, 2013[43]; Speziali et al., 2015[49]). In particular, OA is the result of mechanical and biological events that cause the degradation of articular cartilage. These processes are mediated by excessive synthesis and release of catabolic tissue proteinases, such as matrix metalloproteinases (MMPs), collagenases and aggrecanases, that are upregulated by inflammatory stimuli and oxidative stress, including inflammatory cytokines and mediators, and reactive oxygen species (Goldring and Otero, 2011[15]; Makki and Haqqi, 2015[39]; Lepetsos and Papavassiliou, 2016[35]). Currently, pharmacological treatment for patients with OA is based on some steroidal and non-steroidal anti-inflammatory drugs for alleviation of pain as well as inflammation. However, they can not perfectly prevent the progressive cartilage degradation and repair the impaired cartilage of OA patients, and long-term use of these drugs can lead to severe side effects or toxicity, including gastrointestinal disturbances and cardiovascular risk (Mobasheri, 2013[43]; Goldring and Berenbaum, 2015[14]). Therefore, ideal agents that inhibit cartilage degradation with better safety and efficacy may represent an attractive strategy to treat OA.

Herbal sources have been widely and safely consumed for centuries. Many studies indicate that these herbs have a wide range of diverse biological activities with few side effects (Chen et al., 2015[7]; Hou et al., 2015[18]). Therefore, traditional herbal medicinal sources have been investigated widely as adjuvant therapeutic agents in the treatment of OA (Cameron and Chrubasik, 2013[6]; Dhippayom et al., 2015[10]). Schisandrae Fructus, the dried fruit of *Schisandra chinensis* (Turcz.) Baill. (Magnoliaceae), has been used in traditional medicine for the treatment of various medicinal purposes (Panossian and Wikman, 2008[45]; Chun et al., 2014[9]). The diverse pharmacological effects of Schisandrae Fructus include antioxidant (Kang et al., 2014[23]), anti-tumor (Lv et al., 2015[37]), hepatoprotective (Wat et al., 2016[54]), anti-septic (Kook et al., 2015[30]), neuroprotective (Lee et al., 2012[33]), anti-inflammatory (Bae et al., 2012[3]; Kang et al., 2014[24]), anti-atherosclerotic (Jeong et al., 2015[20]), and anti-atrophic (Kim et al., 2015[27][28]), and anti-diabetic effects (Kwon et al., 2011[31]). Recently we reported that Schisandrae Fructus possessed potential chondroprotective effects of the collagen matrix breakdown in a pro-inflammatory cytokine interleukin (IL)-1β-induced model *in vitro* (Jeong et al., 2015[21]). However, to our knowledge, the in vivo therapeutic effects of this compound and its effects on the molecular mechanisms of OA have not been investigated. Therefore, as a part of our on-going research program for finding novel anti-osteoarthritic substances from traditional medicinal sources, the present study investigated the anti-osteoarthritic potential and underlying mechanism of an ethanol extract of Schisandrae Fructus (SF) in a rat model of metabolic-inhibitor-monosodium iodoacetate (MIA)-induced OA.

## Materials and Methods

### Preparation of SF

The dried fruits of *S. chinensis* were collected from Mungyeong city (Gyeongsangbuk-do, Republic of Korea) and washed three times with tap water before storage at -80 °C. Frozen samples were lyophilized and homogenized using a grinder before extraction with 20 % ethanol at room temperature for 4 h, filtered, and concentrated using a rotary vacuum evaporator (BÜCHI Labortechnik, Flawil, Switzerland). The extract (SF) was dissolved in dimethyl sulfoxide (DMSO; Sigma-Aldrich Chemical Co., St. Louis, MO, USA) as a 50 mg/mL stock solution and stored at 4 °C and diluted with physiological saline to the desired concentration prior to use.

### Animals

Male Sprague-Dawley rats weighing 180~240 g (5 weeks of age) at the start of the experiment were purchased from Samtako Inc. (Osan, Republic of Korea). Two animals were housed per polycarbonate cage in a room under controlled-temperature conditions (20~24 °C, humidity 40~70 %) with controlled lighting (12 h light and/or 12 h dark cycle) and had access to sterile food and R/O water (Lee et al., 2016[34]). This study was carried out in strict accordance with the recommendations of the Guide for the Care and Use of Laboratory Animals of the National Institutes of Health. In addition, the animal protocol used in this study was reviewed by the Dongeui University - Institutional Animal Care and Use Committee on their ethical procedures and scientific care, and it has been approved (Approval Number: A2015-019). 

### Development of OA with MIA injection and administration with SF

The rats were randomized and assigned to treatment groups before the initiation of the study (n = 8 per group). For induction of OA, rats were anesthetized using isoflurane and then given a single injection of 50 μL sterile 0.9 % saline containing 3 mg/kg MIA (Sigma-Aldrich Chemical Co.) using a 0.3 mL insulin syringe (BD Medical-Diabetes Care, Franklin Lakes, NJ, USA) through the patellar ligament into the articular cavity of the right knee. Control rats were injected with an equivalent volume of saline. SF was administered orally once per day for 3 weeks at a dose of 100 mg/kg.

### Measurement of knee joint swelling

After the rats were killed at 3 weeks post-MIA injection, the right knee was isolated, and the femur, tibia, and patella were dissected free of muscle. Knee diameter was measured using a calibrated digital caliper (Mitutoyo, Kawasaki, Japan) to assess the developmental stages of OA on 3 weeks post-MIA injection (Fernihough et al., 2004[12]).

### Serum analysis

At the end of the 3 weeks, the samples of whole blood were collected from the abdominal vein. Blood was allowed to clot for 30 min. Then, the serum was separated via centrifugation at 1,500 g for 10 min and stored at -80 °C. Concentrations of nitric oxide (NO) in the serum samples were determined by measuring nitrite, which is a major stable product of NO, using the Griess reagent. Briefly, 50 μL of serums were mixed with 50 μL of Griess reagent (Sigma-Aldrich Chemical Co.), followed by incubation for 10 min at 37 °C. Optical density was measured at 540 nm using an enzyme-linked immunosorbent assay (ELISA) reader (Dynatech Laboratories, Chantilly, VA, USA) (Kwon et al., 2016[32]). Serum levels of prostaglandin E_2_ (PGE_2_) were determined according to the manufacturer's instructions (ELISA kit, R&D Systems, Minneapolis, MN, USA) (Kim et al., 2016[29]). The pro-inflammatory cytokines including IL-1β, IL-6 and TNF-α, and cartilage degeneration mediators such as cartilage oligomeric matrix protein (COMP) and C-telopeptide of type II collagen (CTX-II) were also determined using ELISA kits (R&D Systems, Minneapolis, MN, USA) according to the manufacturer's recommendations. 

### Joint histological examination

Histological changes were assessed to confirm the effects of SF on cartilage degeneration in the knee joints of MIA-induced OA rats. After the rat sacrifice at 3 weeks, each knee joint was resected, fixed in 10 % formalin (Sigma-Aldrich Chemical Co.) for 24 h at 4° C, and decalcified with 5 % hydrochloric acid (Sigma-Aldrich Chemical Co.) for 4 days at 4° C. After decalcification, the specimens were embedded in paraffin. Sections (2~3 μm) were stained with hematoxylin and eosin (H&E), safranin O-fast green and toluidine blue (Sigma-Aldrich Chemical Co.), respectively, and then observed under Carl Zeiss Axio-plan 2 imaging microscope (Carl Zeiss, Deisenhofen, Germany). All stained slides were histologically evaluated and statistically graded on a scale of 0~13 by double-blind observation, according to the modified Mankin scoring system (Mankin et al., 1971[40]).

### Immunohistochemical analysis

The sections were depleted of endogenous peroxidase activity by treatment with 3 % H_2_O_2_ for 15 min. They were then were blocked with normal goat serum (Sigma-Aldrich Chemical Co.) for 30 min and incubated at 4 °C overnight with primary antibodies followed by the appropriate biotinylated secondary antibodies and horseradish peroxidase-conjugated streptavidin-biotin staining, and finally with a 3,3′-diaminobenzidine (DAKO, Glostrup, Denmark). Primary antibodies against the following proteins were used: inducible NO synthase (iNOS, 1:100; SC-7271, mouse monoclonal, Santa Cruz Biotechnology, Inc., Santa Cruz, CA, USA), cyclooxygenase-2 (COX-2, 1:50; SC-19999, mouse monoclonal; Santa Cruz Biotechnology, Inc.), MMP-13 (1:50; ab51072, rabbit monoclonal; Abcam, Cambridge, UK), COMP (1:50; ab11056, rabbit monoclonal; Abcam) and CTX-II (1:50; PAA686Hu01, rabbit polyclonal; Cloud-Clone Corp., Houston, TX, USA ). Histological changes were examined by a Carl Zeiss Axio-plan 2 imaging microscope (Carl Zeiss) and photographed.

### Statistical analysis

Data were expressed as mean ± standard deviation (SD) for at least three separate determinations for each group. The differences between the groups were examined for statistical significance using the Student's t-test and one-way ANOVA with GraphPad software (GraphPad Inc., La Jolla, CA, USA). A value of p < 0.05 was considered as being significant.

## Results

### SF reduced MIA-induced knee swelling in rats

**Figure 1:**
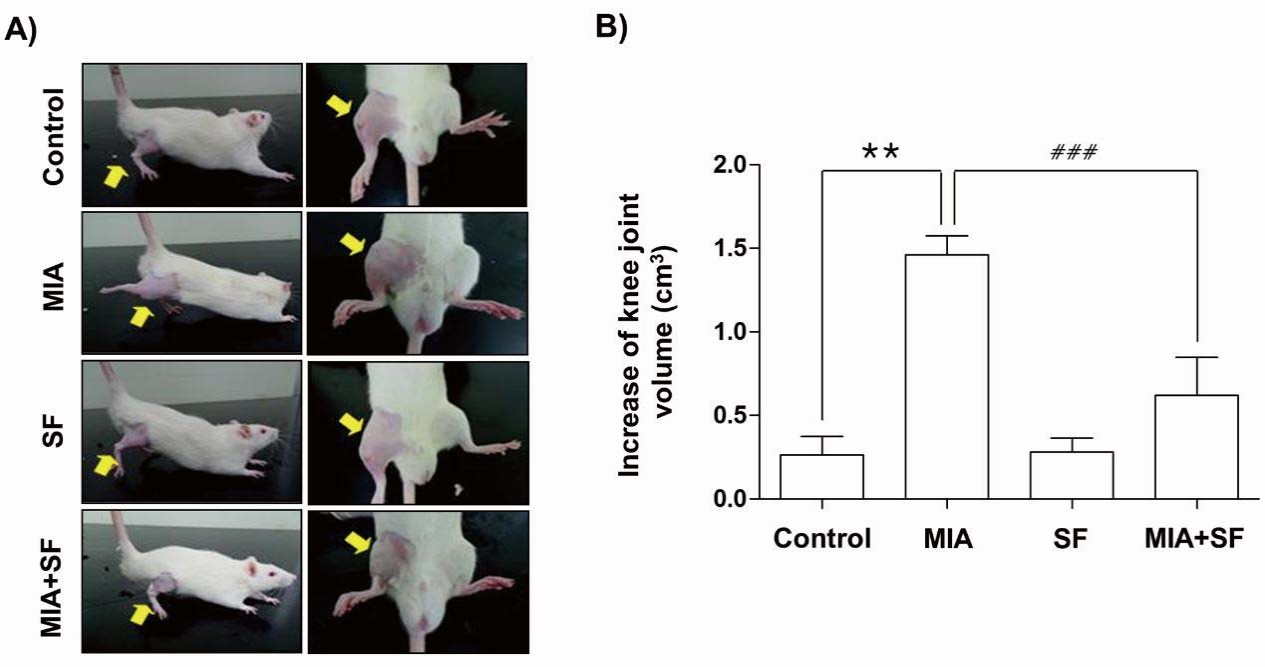
Effects of SF on knee joint swelling in MIA-induced OA rats. (A) Photographs of the hind knee joint at 3 weeks after the model of MIA-induced OA rats. (B) The severity of osteoarthritis during the course of MIA was determined by measuring the volume of the hind knee joint using calibrated digital caliper. Data were expressed as mean ± SD (n = 8) (**p < 0.01 MIA group vs. control group;^ ###^p < 0.001 SF+MIA treatment groups vs. MIA group)

Knee diameters were measured to determine the degree of joint swelling, an index of inflammation that occurred after intra-articular injection of MIA. As shown in Figure 1(Fig. 1), the knee diameter of in MIA groups increased significantly after an intra-articular injection of MIA as compared with normal control group. However, SF treatment significantly reduced the joint swelling at 3 weeks after MIA injection.

### SF ameliorated the histological evaluation of the articular damage in MIA-induced OA rats

**Figure 2:**
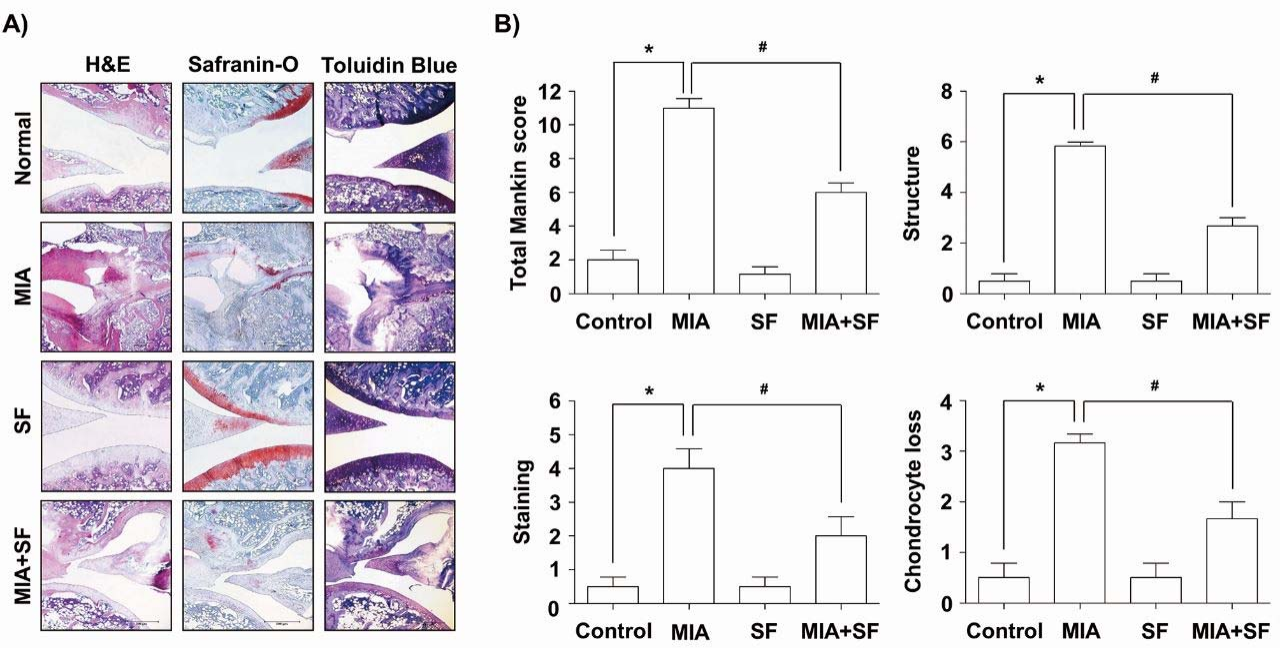
Histological evaluation of joints activity after administration with SF in MIA-induced OA rats. Rats were injected with 3 mg MIA in the right knee. SF was administered orally daily for 3 weeks after MIA injection. (A) The knee joints of OA rats treated with either SF or vehicle control were stained with H&E, Safranin O and toluidine blue. (B) The joint lesions were graded on a scale of 0-13 using the modified Mankin scoring system, giving a combined score for cartilage structure, cellular abnormalities, and matrix staining. Data were expressed as mean ± SD (n = 8) (*p < 0.05 MIA group vs. normal control group;^ #^p < 0.05 SF+MIA treatment groups vs. MIA group)

Because, the cartilage degeneration is the main histologic feature of OA, we investigated the effects of SF on the morphological changes and severity of the particular damage using H&E, Safranin O-fast green, and toluidine blue staining in the MIA-induced OA rat. Our findings showed that MIA-injected group showed the severity of surface irregularity and surface cleft, and matrix loss of articular cartilage associated (Figure 2A(Fig. 2)). However, SF alone had little effect on the structural, morphological changes in the joints, and administration with SF attenuated damages in the articular cartilages compared with those of the MIA-treated group. Therefore, the severity of OA lesion was graded using the modified Mankin scoring system, and we found that the overall modified Mankin's scores were significantly recovered by SF treatment compared with a MIA-treated group (Figure 2B(Fig. 2)).

### SF inhibited the production of pro-inflammatory cytokines in MIA-induced OA rats

**Figure 3:**
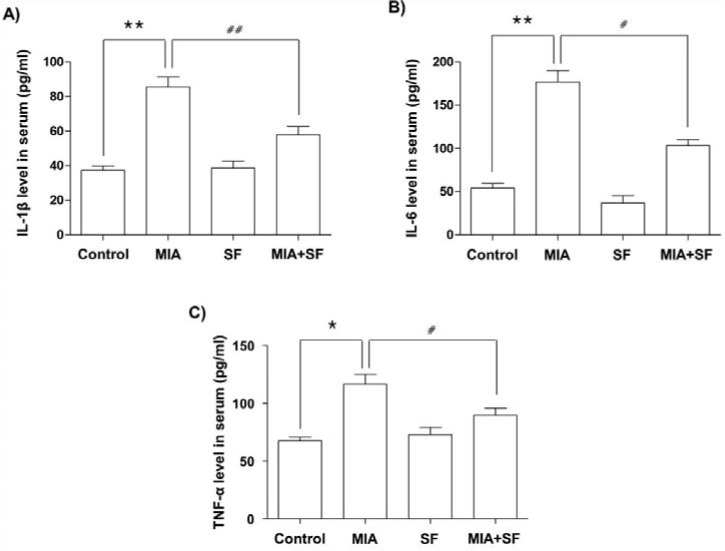
Effects of SF on the cytokines (IL-1β, IL-6, and TNF-α) production in the serum of MIA-induced OA rats. Rats were injected with 3 mg MIA in the right knee. SF was administered orally daily for 3 weeks after MIA injection. (A) The concentrations of IL-1β, (B) IL-6 and (C) TNF-α in serum collected from MIA-induced OA rats treated with or without SF. Data were expressed as mean ± SD (n = 8) (*p < 0.05 and **p < 0.01 MIA group vs. normal control group; ^#^p < 0.05 and ^##^p < 0.01 SF+MIA treatment groups vs. MIA group)

Pro-inflammatory cytokines have important roles in the development and progression of OA (Kapoor et al., 2011[25]; Mabey and Honsawek, 2015[38]). We, therefore, determined whether SF had any inhibitory effects on the production of inflammatory cytokines associated with OA, such as IL-1β, IL-6, and TNF-α. As illustrated in Figure 3(Fig. 3), the serum levels of the cytokines significantly increased in the serum of MIA group compared with those in the saline group; however, the group treated with SF effectively inhibited the production of IL-1β, IL-6, and TNF-α when compared to MIA alone. 

### SF protected the production on NO and PGE_2_, and expression of iNOS and COX-2 in MIA-induced OA rats

**Figure 4:**
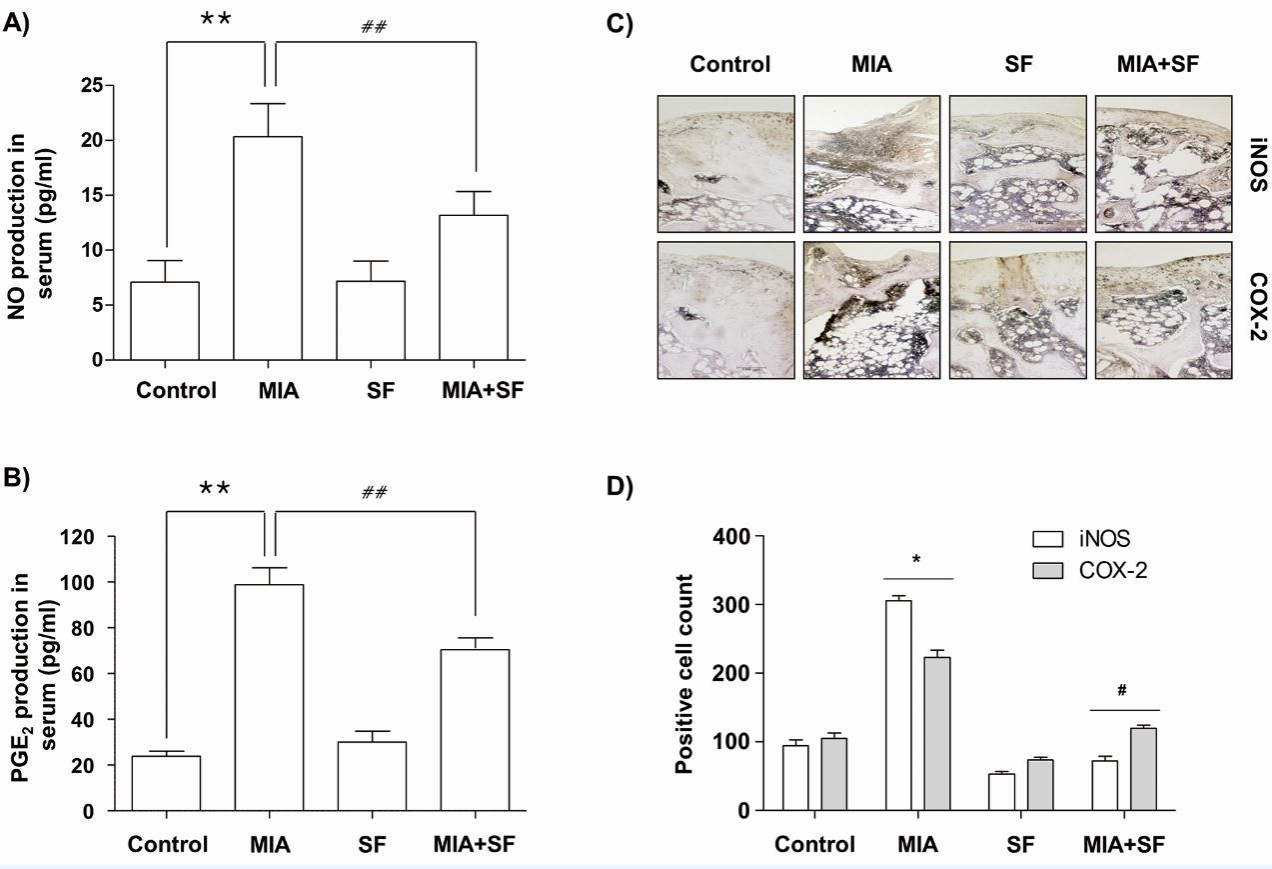
Effects of SF on the production of NO and PGE_2_, and expression of iNOS and COX-2 in MIA-induced OA rats. The contents of NO (A) and PGE_2_ (B) were measured in the serum of MIA-induced OA rat using Griess reaction and commercial ELISA kit, respectively. (C and D) Immunohistochemical staining was used to identify the expression of iNOS and COX-2 in the articular cartilage. Data were expressed as mean ± SD (n = 8) (*p < 0.05 MIA group vs. normal control group; **p < 0.01 MIA group vs. normal control group; ^#^p < 0.05 SF+MIA treatment groups vs. MIA group; ^##^p < 0.01 SF+MIA treatment groups vs. MIA group)

Because overproductions of pro-inflammatory mediators such as NO and PGE_2_ also have been correlated to the pathophysiology of OA (Amin et al., 2000[1]; Martel-Pelletier et al., 2003[42]), we investigated the effects of SF on NO and PGE_2_ release in MIA-induced OA rats. As indicated in Figure 4A and B(Fig. 4), a substantial increase in the NO and PGE_2_ levels were detected in MIA-induced OA rats when compared to normal control group; however, these levels were significantly decreased following SF administration. We subsequently investigated whether the inhibitory effects of SF on NO and PGE_2_ production were related to the regulation of the expression of their synthesis enzymes, iNOS, and COX-2, in knee joints by immunohistochemical analysis. The results showed that compared to the control group, MIA remarkably induced the expression of iNOS and COX-2, while, SF obviously suppressed elevated expression levels of iNOS and COX-2 in the cartilaginous tissues of the MIA-induced OA rats (Figure 4C and D(Fig. 4)). 

### SF down-regulated the MMP-13 expressions in MIA-induced OA rats

**Figure 5:**
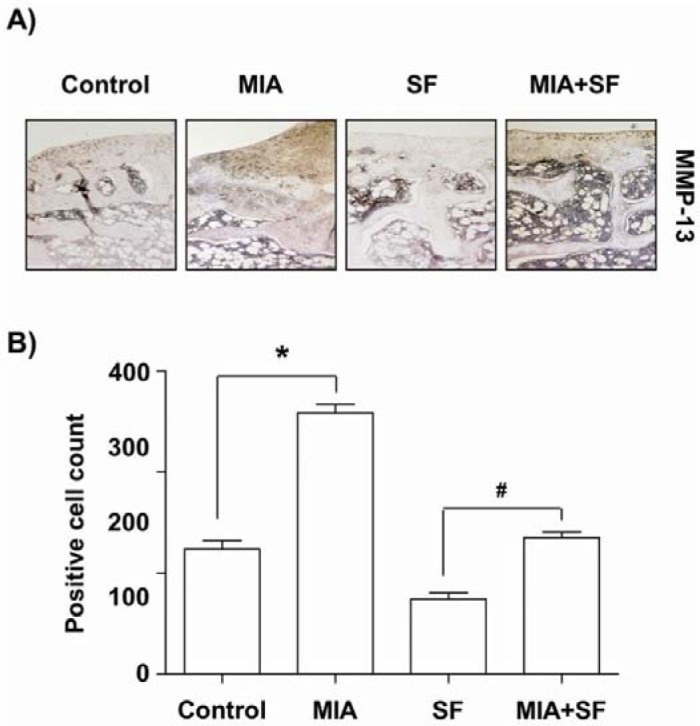
Effects of SF on the expression of MMP-13 in MIA-induced OA rats. (A) The immunohistochemical staining was used to identify the expression of MMP13 in the articular cartilage. (B) Data were expressed as mean ± SD (n = 8) (*p < 0.05 MIA group vs. normal control group; ^#^p < 0.05 SF+MIA treatment groups vs. MIA group)

The progressive destruction of cartilage degradation in OA is caused by several matrix-degrading enzymes including MMPs produced by the chondrocytes and synovium (Burrage et al., 2006[5]; Troeberg and Nagase, 2012[51]). Because, among the MMPs family, MMP-13 play a most important role in degrading cartilage (Takaishi et al., 2008[50]; Li et al., 2011[36]), we examined the effects of SF on the expression of MMP-13 in MIA-stimulated OA cartilage. Our results demonstrated that the number of chondrocytes staining positive for MMP-13 was increased after MIA injection, with significantly lower percentages of MMP-13-positive chondrocytes evident in the SF-administered group relative to saline-treated controls (Figure 5(Fig. 5)).

### SF diminished the production of COMP and CTX-II in MIA-induced OA rats

**Figure 6:**
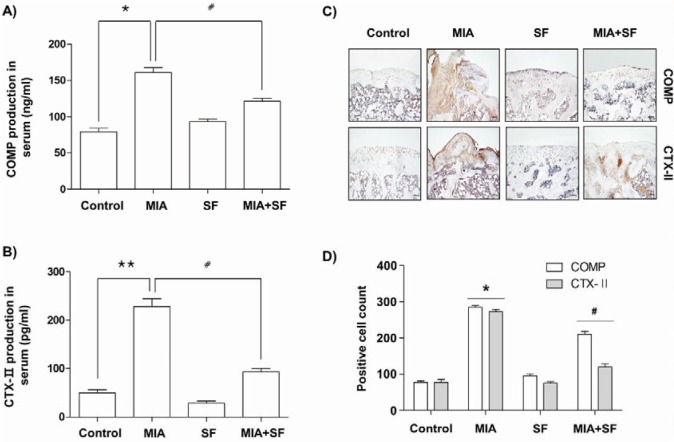
Effects of SF on production and expression of COMP and CTX-Ⅱ in MIA-induced OA rats. (A and B) The COMP and CTX-II production were measured in the serum of MIA-induced OA rat using a commercial ELISA kits. (C and D) Immunohistochemical staining was used to identify the expression of COMP and CTX-II in the articular cartilage. Data were expressed as mean ± SD (n = 8) (*p < 0.05 MIA group vs. normal control group; **p < 0.01 MIA group vs. normal control group; ^#^p < 0.05 SF+MIA treatment groups vs. MIA group)

We next investigated the effects of SF on the levels of COMP and CTX-II, well-established biomarkers for diagnosis and OA progression (Goode et al., 2012[16]; Saberi Hosnijeh et al., 2016[48]), which play key roles during subsequent progressive osteophytes in multiple knee compartments. Our ELISA data indicated that excessive production of COMP and CTX-II were evident in the serum of MIA-induced OA rats; these levels were significantly diminished following SF administration (Figure 6A and B(Fig. 6)). Consistent with the results of ELISA, immunohistochemistry assay showed that oral administration with SF caused a significant decrease in expression of COMP as well as CTX-II compared with that in the normal control group (Figure 6C and D(Fig. 6)).

## Discussion

In this study, we investigated whether or not SF exerts a chondroprotective effect in a rat OA model by injection with MIA, which is known to induce OA through interruption of chondrocyte metabolism (Barve et al., 2007[4]). Our data showed that oral administration of SF led to a significant decrease of structural changes such as joint space narrowing and cartilage destruction in MIA-induced OA rats, which was associated with a reduction of pro-inflammatory molecules, MMP-13 and both biomarkers of cartilage and bone metabolism such as COMP and CTX-II. 

The compelling evidence demonstrated that pro-inflammatory cytokines are significantly elevated in synovial fluid from OA patients and play critical roles in the promotion of the catabolic processes in OA, causing cartilage degradation (Kapoor et al., 2011[25]; Rahmati et al., 2016[47]). High levels of pro-inflammatory cytokines have been found in synovial fluid from OA patients and several models of cartilage degradation (Goldring and Otero, 2011[15]; Kellesarian et al., 2016[26]). Among these cytokines, IL-1β is highly over-expressed in the cartilage as well as in the synovial tissue while the expression of IL-1Rα, a receptor antagonist of the IL-1 family (Jotanovic et al., 2012[22]). This cytokine inhibits proliferation and triggers apoptosis of chondrocytes and blocks the extracellular matrix (ECM) structural compounds synthesis by activating MMPs including MMP-13 (Mabey and Honsawek, 2015[38]; Rahmati et al., 2016[47]). IL-6 has also been reported to act as one of the main pro-inflammatory cytokines involved in the pathophysiology of OA. IL-6 induces destruction of joint and cartilage by stimulating the activation of osteoclasts and differentiation of mesenchymal cells into chondroblasts (Doss et al., 2007[11]; Jotanovic et al., 2012[22]). In addition, previous studies revealed that TNF-α has similar to or synergistic with IL-1β and IL-6 in the production of matrix-degrading enzymes and inhibition of proteoglycan synthesis, resulting in loss of cartilage and bone resorption during the process of OA development (Jotanovic et al., 2012[22]; Kellesarian et al., 2016[26]). In accordance with, many studies demonstrated that anti-inflammatory agents capable of inhibiting the production of those cytokines may have the potential to control or treatment of OA (Kapoor et al., 2011[25]; Mabey and Honsawek, 2015[38]). Hence, we investigated the anti-inflammatory effects of SF by measurements of the serum levels of pro-inflammatory cytokines, such as IL-1β, IL-6, and TNF-α, in MIA-induced OA rats, and found that SF administration decreased these cytokines, the MIA increased these parameters. Taken together, the present results indicate that SF has a potential of prevention against inflammatory responses, and subsequently might reduce the damage of articular cartilage.

In addition to the roles of inflammatory cytokines, pro-inflammatory mediators such as NO and PEG_2_ plays an extremely important role in the development of inflammation in OA (Amin et al., 2000[1]; Rahmati et al., 2016[47]). These pro-inflammatory mediators can induce cell death of chondrocytes and loss of cartilage matrix in the pathogenesis of OA, and they are also significantly elevated in cartilage and synovial tissues from OA patients (Notoya et al., 2000[44]; Park et al., 2006[46]). Moreover, the pro-inflammatory cytokines can stimulate the production of pro-inflammatory mediators, such as NO and PEG_2_, through activation of chondrocytes (Martel-Pelletier et al., 2006[41]; Rahmati et al., 2016[47]). Therefore, we next investigated the effects of SF on the release of NO and PEG_2_ in MIA-induced OA rat model. Our results clearly demonstrated that the serum levels of NO and PEG_2 _were significantly higher in the MIA group compared with the control group. However, SF effectively reduced MIA-induced elevation of NO and PEG_2_ production by suppressing upstream molecules iNOS and COX-2 expression, consistent with a previous our report that SF reduced production of NO and PEG_2_ in IL-1β-stimulated human chondrocytes (Jeong et al., 2015[21]) and lipopolysaccharide-activated murine macrophage (Kang et al., 2014[24]). 

Accumulated evidence suggested that MMPs are important metalloproteases involved in tissue remodeling including the turnover, catabolism, and degradation of the ECM (Burrage et al., 2006[5]; Troeberg and Nagase, 2012[51]). MMPs expression could be up-regulated by pro-inflammatory cytokines in a variety of tissues and cell types, including articular chondrocytes (Goldring and Otero, 2011[15]; Takaishi et al., 2008[50]). Among the MMPs, MMP-13 is critical for degrading collagens, proteoglycans and other ECM macromolecules in the osteoarthritic pathological process (Takaishi et al., 2008[50]; Li et al., 2011[36]). We then analyzed the expression of MMP-13, to assess the effects of SF on the catabolic activity of chondrocytes. Consistent with previous studies (Andereya et al., 2006[2]; Barve et al., 2007[4]), the serum levels of MMP-13 and the percentages of MMP-13-positive chondrocytes were significantly higher in the MIA-treated group than in the saline-treated controls. However, our results demonstrated that the SF-administrated rats had fewer MMP-13-producing cells than did the MIA-treated rats, which was connected with lowering MMP-13 production. These observations support the fact that SF might have a chondroprotective effect by reducing the production and activation of MMP-13.

Several clinical studies in OA patients and OA animal models demonstrated that the elevated levels of COMP and CTX-II are correlated with increased risk and progression of OA. COMP, a pentameric glycoprotein, is one of the essential components of the extracellular matrix of the cartilage (Goode et al., 2012[16]; Saberi Hosnijeh et al., 2016[48]). COMP functions as a regulator in governing the assembly of type II collagen fibers in cartilage, thereby this glycoprotein stabilizes the collagen network in cooperation with other matrix proteins (Christgau et al., 2001[8]). However, its levels during the development of OA and under inflammatory condition are obviously increased in serum and synovial fluid and positively correlated with joint damage in knee OA (Vilím et al., 2002[53]; Verma and Dalal, 2013[52]). In addition, CTX-II is produced by degradation of type II collagen through the action of proteases with cartilage injury or degeneration and finally excreted in the urine (Christgau et al., 2001[8]; Freeston et al., 2011[13]). CTX-II contents were also elevated in OA patients as compared with normal individuals, which levels are associated with both the prevalence and progression of OA (Jansen et al., 2009[19]; Freeston et al., 2011[13]). These observations indicated that these two factors have the potential to be prognostic biomarkers for monitoring cartilage degradation in patients with OA (Goode et al., 2012[16]; Saberi Hosnijeh et al., 2016[48]). In our ELISA study, both serum COMP and CTX-II levels in the MIA-induced OA group were highly increased compared to the control group; however, treatment with SF significantly prevented the increase. In agreement with the result, the immunohistochemistry data also showed that rats injected with MIA dramatically increased COMP and CTX-II expression in articular cartilage, and besides administration with SF significantly reduced their expression in MIA-induced OA rats. Therefore, it is possible that the reduction in COMP and CTX-II serum levels by SF most likely represents suppressed MIA-induced degradation of cartilage, as cartilage is a major contributor to circulating COMP and CTX-II levels.

## Conclusions

In conclusion, we demonstrated that administration with SF effectively attenuated the severity of articular cartilage destruction in MIA-induced OA of the knee joint in rats. To the best of our knowledge, this is the first report to demonstrate the antiarthritic effects of SF on MIA-induced OA model. The antiarthritic effects of SF were associated with the decreased production of pro-inflammatory cytokines, such as IL-1β, IL-6 and TNF-α, and mediators including NO and PGE_2 _through reducing their corresponding genes expression. SF also protected the articular cartilage damage by suppression of MMP-13 and two representative biomarkers for diagnosis of OA, COMP, and CTX-II, in the OA animal model induced by MIA. Based on the results of this study, we suggest that Schisandrae Fructus has excellent potential as a therapeutic modality for treating OA. 

## Conflicts of interest

The authors declare that there is no conflict of interest.

## Acknowledgement

This work was supported by the High Value-added Food Technology Development Program (314043-3), Ministry of Agriculture, Food and Rural Affairs, Republic of Korea
